# Time to onset and duration of botulinum toxin efficacy in movement disorders

**DOI:** 10.1007/s00415-022-10995-2

**Published:** 2022-02-03

**Authors:** Claudia Ledda, Carlo Alberto Artusi, Antonella Tribolo, Domiziana Rinaldi, Gabriele Imbalzano, Leonardo Lopiano, Maurizio Zibetti

**Affiliations:** 1 Department of Neuroscience “Rita Levi Montalcini”, University of Turin, Via Cherasco 15, 10126 Turin, Italy; 2 Neurology 2 Unit, A.O.U. Città della Salute e della Scienza di Torino, Corso Bramante 88, 10126 Turin, Italy; 3 Dipartimento di Neuroscienze, Salute Mentale e Organi di Senso, Sapienza Università di Roma, Via di Grottarossa, 1035, 00189 Rome, Italy

**Keywords:** Botulinum toxin, Efficacy, Duration, Movement disorders, Sialorrhea

## Abstract

**Background:**

Botulinum toxin (BoNT) is a valuable treatment in movement disorders; however, time to onset and duration of efficacy may widely differ among patients. We aimed to clarify the impact of main demographic and clinical features on time to onset and duration of BoNT efficacy.

**Methods:**

We analyzed time-to-onset and duration of BoNT efficacy in 186 consecutive patients treated with BoNT for blepharospasm, cervical dystonia, facial hemispasm, oromandibular dystonia, limb dystonia, and sialorrhea due to Parkinsonism. The following factors were considered as potential efficacy predictors: doses and types of toxin, sex, age, years of treatment, and clinical condition. Kruskall–Wallis, Spearman correlation, and multivariate linear regression were used for statistical analysis.

**Results:**

The average time to onset was 6.7 ± 5 days and duration of BONT efficacy 78.5 ± 28.4 days. Both time to onset and duration of efficacy were correlated with BoNT doses (*p*: 0.007 and *p*: 0.02). The multiple regression analysis showed that sex, age, years of BoNT treatment, doses, type of toxin, and clinical condition significantly predicted time to onset (*F*(11, 171) = 2.146, *p*: 0.020) with age being the strongest predictor (*p*: 0.004).

The same model explained 20.1% of the variance of duration of BoNT efficacy, showing a significant prediction of the outcome (*F*(11, 164) = 3.754, *p* < 0.001), with doses (*p* < 0.001), type of toxin (*p*: 0.017), and clinical condition (*p* < 0.001) being the strongest predictors.

**Conclusion:**

Our findings suggest that age, type of toxin, clinical condition and especially doses may account for the variability of BoNT efficacy in terms of time to onset and duration.

**Supplementary Information:**

The online version contains supplementary material available at 10.1007/s00415-022-10995-2.

## Introduction

Botulinum toxin (BoNT) is one of the most potent biological toxins and has emerged as a valuable and versatile therapeutic agent for many neurological applications [1].

BoNT acts by inhibiting the release of acetylcholine from the presynaptic terminal at the neuromuscular junction, leading to inhibition of neurotransmitter release and therefore temporary weakness of the target muscle [1, 2]. Paresis is typically expected to occur after 2–5 days from injections, reaches its peak at 5–6 weeks and lasts for 2–3 months [3].

There are three main BoNT type A preparations used in Europe in clinical practice: onabotulinumtoxinA (onaBoNT-A), abobotulinumtoxinA (aboBoNT-A) and incobotulinumtoxinA (incoBoNT-A) [4, 5]. These three formulations are considered to have similar efficacy and safety profile, but different excipients and diluents are used for each preparation of the commercial vial so that the potency of the units of the three formulations do not have a comparable effect in clinical practice. Generally, it is estimated that 1 U of onaBoNT-A corresponds to 1 U of incoBoNT-A and 3–5 U of aboBoNT-A [3, 5].

Time to onset and duration of efficacy of BoNT may vary widely among patients and differences may be related to genetics, target muscles (mass, size, thickness, depth below the skin), units injected and technique of injection [3, 6]; however, data are missing regarding the influence that clinical-demographic data, type of BoNT, indication for which BoNT is used, and doses utilized may have. Also, only few data are available on time to onset of BoNT effect, since the vast majority of studies on BoNT focused on its efficacy duration.

In this study, we aimed to investigate the time to onset and duration of BoNT efficacy in a large sample of patients affected by movement disorders, analyzing the impact of demographic and clinical features, and the role of different types of BoNT, dosages, and clinical conditions.

## Methods

We conducted an observational study enrolling all consecutive patients attending the Botulinum toxin outpatient clinic of ‘Città della Salute e della Scienza di Torino’, Molinette Hospital, University of Torino, between May, 1 2020 and May, 31 2021. Inclusion criteria were: (1) attending Botulinum toxin outpatient clinic for dystonia, hemifacial spasm or sialorrhea due to parkinsonism; (2) having received at least two courses of BoNT injection; (3) being able to reliably answer to our questionnaire. All patients were treated by two movement disorder experts using the same injection technique.

When attending the clinic, each patient was asked to answer a structured questionnaire related to the previous BoNT treatment (Supplementary material). The following data were collected by the questionnaire and the informatic health records: sex, age, type of disease, disease duration, years of BoNT treatment, type (onaBoNT-A, aboBoNT-A or incoBoNT-A) and total dose of BoNT used at the last injection, number of days between BoNT injection and onset of clinical effect firstly noticed by the patient, and duration of BoNT efficacy, defined as the time until the patient started perceiving the toxin’s effect was wearing off [5]. BoNT doses were compared with the assumption that 1 U of onaBoNT-A corresponds to 1 U of incoBoNT-A and 3 U of aboBoNT-A [3, 5].

The local ethical committee ‘Comitato Etico Interaziendale AOU Città della Salute e della Scienza di Torino’ approved the study and written informed consent was obtained by enrolled patients.

### Statistical analysis

Demographic and clinical features were summarized as mean ± standard deviation or percentages, as appropriate. Kruskall–Wallis was used for the comparison between different conditions for which the patients were treated to analyze the differences regarding time to onset and duration of BoNT efficacy. Spearman correlation was used for the analysis of correlation between BoNT doses and average time to onset and duration of BoNT efficacy, independently from the condition. Two multivariate linear regression tests were used to analyze the association between time to onset (first test) and duration of BoNT efficacy (second test) as dependent variables and the following independent variables: age, sex, doses, years of treatment with BoNT, clinical condition, and type of toxin. All demographic and clinical independent variables included in the multivariate linear regression tests were chosen if they demonstrated either a *p* value < 0.1 at a preliminary univariate logistic regression analysis or possible clinical relevance.

Finally, two covariance analyses were run with type of condition as categorical independent variable and age, doses, and type of toxin as covariates; the first analysis used time to onset and the second analysis duration of BoNT efficacy as dependent variable.

## Results

We enrolled 186 patients treated with BoNT for the following conditions: 34.9% (*n* = 65/186) for blepharospasm, 24.7% (*n* = 46/186) for cervical dystonia, 21.5% (*n* = 40/186) for hemifacial spasm, 7.5% (*n* = 14/186) for sialorrhea due to parkinsonism, 8.6% (*n* = 16/186) for focal or segmental limb dystonia, and 2.7% (*n* = 5/186) for oromandibular dystonia. The main demographic and clinical characteristics of patients are reported in Table 1. Thirty-one patients were treated with incoBoNT-A, 98 with onaBoNT-A and 57 with aboBoNT-A (Table 1).

**Table 1:** Main clinical and demographic characteristics of patients included in the study

	Total sample (*n* = 186)	Blepharospasm (*n* = 65)	Cervical dystonia (*n* = 46)	Hemifacial spasm (*n* = 40)	Oromandibular dystonia (*n* = 5)	Focal dystonia (*n* = 16)	Sialorrhea (*n* = 14)
Sex							
Female	115 (61.8%)	46 (70.8%)	27 (58.7%)	28 (70%)	3 (60%)	8 (50%)	3 (21.4%)
Male	71 (38.2%)	19 (29.2%)	19 (41.3%)	12 (30%)	2 (40%)	8 (50%)	11 (78.6%)
Age							
	68.2 ± 15 (20–96)	73.7 ± 12.2 (26–96)	61 ± 15.1 (20–88)	72.6 ± 13.3 (43–91)	55.9 ± 8 (49–68)	54.7 ± 17.6 (24–83)	74.9 ± 8.1 (56–85)
Years of treatment with BoNT							
	8.7 ± 7.2 (1–33)	9.1 ± 6.3 (1–31)	10 ± 7.4 (1–27)	11.4 ± 8.9 (1–33)	5.4 ± 2 (2–7)	4.1 ± 3.2 (1–14)	2.7 ± 1.4 (1–5)
Type of BoNT							
OnaBoNTA	98 (52.7%)	52 (80%)	1 (2.2%)	32 (80%)	1 (20%)	0	12 (85.7%)
IncoBoNTA	31 (16.7%)	13 (20%)	6 (13%)	7 (17.5%)	2 (40%)	1 (6.2%)	2 (14.3%)
AboBoNTA	57 (30.6%)	0	39 (84.8%)	1 (2.5%)	2 (40%)	15 (93.8%)	0
Dose of BoNT (U)							
	63 ± 55.4 (2.5–500)	45 ± 19.7 (10–95)	109 ± 46.9 (33.3–266.7)	31.1 ± 18.8 (7.5–65)	59 ± 55.3 (6.7–133.3)	78.2 ± 62 (16.7–266.7)	50.7 ± 19 (20–90)

Data are reported as mean ± standard deviation (range) or number of patients (percentage)

*BoNT* botulinum toxin

Considering all patients, the average time to onset of efficacy was 6.7 days ± 5 (range 1–30) and duration of BoNT efficacy 78.5 days ± 28.4 (range 15–180). Average time to onset and duration of BoNT efficacy in patients treated for blepharospasm were respectively 5.7 days ± 3.9 (range 1–15) and 73.3 days ± 26.9 (range 15–165), for cervical dystonia 7.3 days ± 4.8 (range 1–21) and 81.2 days ± 26.4 (range 25–150), for hemifacial spasm 6.7 days ± 5.3 (range 1–25) and 81 days ± 30.6 (range 21–165), for sialorrhea 6.2 days ± 4.4 (range 2–15) and 71.4 days ± 30.2 (range 30–120), for focal limb dystonia 7.7 days ± 6.8 (range 1–25) and 87.2 days ± 33.9 (range 30–180), and for oromandibular dystonia 10.4 days ± 11 (range 3–30) and 90 days ± 18.4 (range 75–120).

Comparing patients per clinical condition, we found significant difference for BoNT doses but not for time to onset and duration of BoNT efficacy. Independently from the condition, both time to onset and duration of BoNT efficacy were significantly correlated with doses (*p*: 0.007 and *p*: 0.02). The multiple regression analysis showed that sex, age, years of BoNT treatment, doses, type of toxin, and clinical condition explained 12.1% of BoNT time to onset variance. The model significantly predicted time to onset (*F*(11, 171) = 2.146, *p*: 0.020) with age being the strongest predictor (*p*: 0.004) (Table 2).

**Table 2:** Multiple regression analysis of time to onset of BoNT effect

Model summary	*R*	*R*^2^	Adjusted *R*^2^	Standard error	Durbin–Watson
	0.348	0.121	0.065	4,88,630	2037

ANOVA	Sum of squares	Degrees of freedom	Mean squares	*F*	*p* value
Regression	563,539	11	51,231	2146	0.020*
Residual	4082,789	171	23,876		
Total	4646,328	182			

Covariance analysis	*B*	Standard error	Beta	*t*	*p* value
Doses	0.018	0.011	0.163	1.670	0.097
Years of treatment	− 0.063	0.058	− 0.090	− 1.085	0.279
Age	− 0.084	0.029	− 0.249	− 2.879	0.004*
Sex	0.401	0.796	0.039	0.504	0.615
IncobotulinumtoxinA	− 0.224	1.117	− 0.017	− 0.200	0.842
AbobotulinumtoxinA	− 2.225	1.866	− 0.204	− 1.192	0.235
Cervical dystonia	1.389	1.919	0.120	0.723	0.470
Hemifacial spasm	1.401	1.025	0.113	1.367	0.173
Oromandibular dystonia	3.699	2.483	0.120	1.490	0.138
Focal dystonia	1.721	2.265	0.096	0.760	0.448
Sialorrhea	0.273	1.590	0.014	0.172	0.864

As a categorical, non-dichotomous variable, type of toxin was inserted in the statistical model as a dummy variable with onabotulinumtoxin-A as the reference level (thus not present in the table)

Likely, type of disease was inserted as a dummy variable with blepharospasm as the reference level (thus not present in the table)

*BoNT* botulinum toxin

The same model explained 20.1% of the variance of duration of BoNT efficacy, showing a statistically significant prediction of the outcome (*F*(11, 164) = 3.754, *p* < 0.001), with doses (*p* < 0.001), type of toxin (*p*: 0.017), and clinical condition (*p* < 0.001) being the strongest predictors (Table 3). Covariance analysis confirmed that the type of condition for which the patient is treated is highly significant in determining duration of BoNT efficacy (*p* < 0.001), independently from age, doses, and type of toxin. This analysis showed that blepharospasm had a significantly shorter duration of BoNT efficacy than cervical dystonia (*p* < 0.001) and focal limb dystonia (*p*: 0.003); cervical dystonia a significantly longer duration than hemifacial spasm (*p*: 0.006) and sialorrhea (*p*: 0.006); hemifacial spasm a significantly shorter duration than focal limb dystonia (*p:* 0.021); focal limb dystonia a significantly longer duration than sialorrhea (*p*: 0.017).

**Table 3:** Covariance analysis of duration of BoNTa efficacy

Model summary	*R*	*R*^2^	Adjusted *R*^2^	Standard error	Durbin–Watson
	0.449	0.2013	0.148	2,621,350	0.387

ANOVA	Sum of squares	Degrees of freedom	Mean squares	*F*	*p* value
**Regression**	283,77	11	2579,785	3754	< 0.001*
**Residual**	112,692,227	164	687,148		
**Total**	141,069,858	175			

	*B*	Standard error	Beta	*t*	*p* value
Doses	− 0.259	0.059	− 0.409	− 4.369	< 0.001*
Years of treatment	− 0.390	0.319	− 0.100	− 1.224	0.223
Age	0.299	0.158	0.159	1.895	0.060
Sex	− 1.261	4.357	− 0.022	− 0.289	0.773
IncobotulinumtoxinA	− 14.766	6.112	− 0.196	− 2.416	0.017*
AbobotulinumtoxinA	− 8.999	10.071	− 0.147	− 0.894	0.373
Cervical dystonia	34.674	10.334	0.530	3.355	< 0.001*
Hemifacial spasm	5.649	5.633	0.080	1.003	0.317
Oromandibular dystonia	30.590	13348	0.180	2292	0023*
Focal dystonia	32.156	12.226	0.327	2.630	0.009*
Sialorrhea	− 5.314	9.164	− 0.045	− 0.580	0.563

As a categorical, non-dichotomous variable, type of toxin was inserted in the statistical model as a dummy variable with onabotulinumtoxin-A as the reference level (thus not present in the table)

Likely, type of disease was inserted as a dummy variable with blepharospasm as the reference level (thus not present in the table)

*BoNT* botulinum toxin

## Discussion

In our study on 186 patients treated with BoNT for movement disorders or sialorrhea, we observed a high variability in time to onset and duration of BoNT efficacy. Our analyses showed that such a variability can be predicted by specific demographic and clinical characteristics. In particular, we observed that the total BoNT dose injected is significantly associated with the time to onset and duration of efficacy, independently from the underlying disease. However, in the multivariate analysis, time to onset of BoNT efficacy was mainly predicted by age, with older patients showing a shorter time to achieve benefit from treatment. The duration of BoNT efficacy was predicted by type of toxin, doses, and underlying clinical condition.

While previous data already analyzed the duration of BoNT efficacy for different diseases [7–10], the literature is less informative about the time to onset of BoNT efficacy and its modifiers.

Among the few studies which analyzed time to onset of BoNT efficacy for movement disorders, an average time of 5.4 days for facial hemispasm [11], 7.1 days for blepharospasm [12], and 6.1 days for different movement disorders was observed, in the absence of an influence of the type of toxin [11–13]. Considering blepharospasm, facial hemispasm, cervical dystonia, focal limb dystonia, oromandibular dystonia, and sialorrhea, we found an average of 6.7 days of time to onset of BoNT efficacy, which is consistent with previous findings. Moreover, our analysis on factors influencing the variability of outcome showed that age is inversely correlated with time to onset (i.e., time to onset is shorter in older patients), even when controlling for sex, years of BoNT treatment, doses, type of toxin, and clinical condition. This finding could be explained by the muscular changes in aging, related to the increasing percentage of type I fibers compared to type II and the progressive loss and enlargement of active motor units [14]. Similarly, the proportional volume of fat and fibrovascular tissue increases in parotid glands with age, with a reduction of the volume of acini, the functional part of the gland [15].

**Fig. 1:**
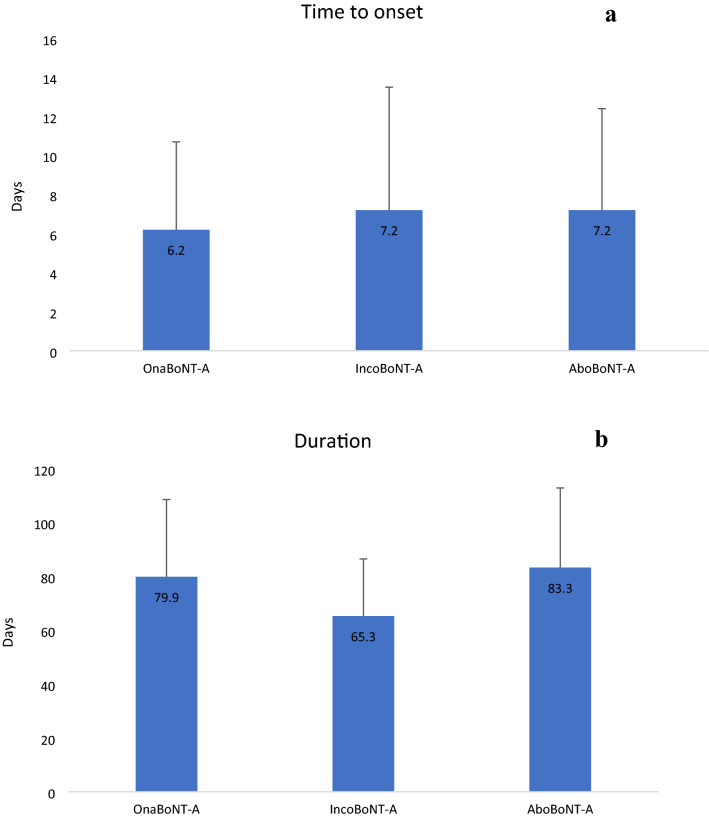
BoNTa efficacy in different types of toxins. **a** Days between BoNT injection and perceived improvement of symptoms. **b** Days between BoNT injection and perceived wearing off of the effect. ^*a*^*BoNT* botulinum toxin

Literature data seem to suggest that the duration of neuromuscular blockade differs according to the BoNT serotypes [16], but clinical comparisons on injected patients reported mixed results. Some studies revealed a similar duration of effect of BoNT-A formulations [7, 10, 12]; others found conflicting results, with longer efficacy duration of onaBoNT-A vs. aboBoNT-A in patients treated for cervical dystonia, hemifacial spasm and blepharospasm and longer efficacy duration of aboBoNT-A vs. onaBoNT-A for hemifacial spasm [11, 17]. A crossover study investigating the use of ona- and aboBoNT-A in cervical dystonia, blepharospasm and hemifacial spasm, with dose ratios of 5:1 and 4:1, found that duration of BoNT efficacy was longer with onaBoNT-A [18]. A similar study conducted with patients treated for cervical dystonia with ona- and aboBoNT-A at a ratio of 3:1 suggested a similar effect duration [7]. Our analysis revealed that the type of toxin did not influence time to onset but influenced duration of BoNT efficacy, with onaBoNT-A achieving a longer duration of efficacy than incoBoNT-A independently from clinical condition and doses (Fig. 1). At a conversion rate of 3:1, aboBoNT-A did not reveal any significant difference when compared with onaBoNT-A.

The extent of paresis provoked by the botulinum toxin is correlated to the dose, but it isn’t clear if the dose affects duration of action. It has been proposed that when lower doses of BoNT are used, duration correlates with the amount injected, while duration saturates around 3 months when higher doses are used [3]. Poewe et al., in 1998, conducted a study with the objective to analyze the dose–response correlation in a group of 75 treatment-naïve patients affected by cervical dystonia. Results demonstrated a positive dose–response correlation for the magnitude and duration of improvement, but also for the occurrence of adverse events [19]; however, in another study conducted in 2016 they reported that duration of treatment did not change, regardless of the dose used [20]. Li et al. compared high versus low doses of BoNT (25 U vs 50 U) in patients with hemifacial spasm and time to onset didn’t differ significantly, but duration of efficacy was longer with the higher dose [21]. A review conducted by Flynn and coll., examining duration of effect of botulinum toxin for facial aesthetic applications, concluded that dose-duration relationships are not robust and require additional investigations [22]. Differently from previous studies, we found that doses are relevant in determining the duration of BoNT efficacy, with higher doses correlated to a lower duration of efficacy. It can be hypothesized that patients receiving higher doses of BoNT were those patients with a more severe disease, for whom a higher dosage was applied due to early recurrence of symptoms.

It is important to highlight that although we explored the main clinical and demographic predictors, the multivariate model explained a significant yet small part of the outcome variability (i.e., 12% and 20%). This finding could be explained by the fact that other relevant features, such as target muscles, variability of injection between patients, depth of muscle below the skin, heterogeneity in disease severity, recall bias, and toxin dilution, should be considered as possible modifiers in the BoNT outcome.

The strength of our findings is tempered by some limitations. First of all, the main outcomes are based on a patient’s self-assessment of their symptoms, rather than objective scale-based measures. The clinical ground of the study and the inclusion of different movement disorders led us to opt for such a subjective evaluation, which is typically used during clinical practice.

Furthermore, ambiguity may exist regarding the exact definition of treatment time to onset and duration of BoNT efficacy according to subjects and underlying clinical conditions. On the other hand, the literature does not provide specific indications on the best way to assess BoNT efficacy in terms of onset and duration, with many not-validated methods proposed, such as the diminished muscle activity assessed either by the physician or the patient [3]. The main parameter used in the literature to evaluate the duration of efficacy of BoNT is the duration of clinical response, but many surrogates, as duration of peak to benefit, the last moment after treatment in which a difference in muscle lengthening can be detected, the relapse rate (time to return to baseline level) and time between injections, have been used, accounting for the variability of findings [5, 22]. Finally, we decided to include in the analyses the five patients with oromandibular dystonia; however, we acknowledge that the sample size for this category of patients is probably too small to obtain significant results. We also performed the multivariate analyses excluding this subgroup of five patients without differences in *p* values and *R*^2^ of the total model (data not presented).

Taking into account the above-mentioned limitations, our data provide useful information on clinical and demographic data influencing two relevant patient-centered parameters of BoNT efficacy in different movement disorders. In particular, we found that: (i) sex, age, years of BoNT treatment, doses, type of toxin, and clinical condition are relevant in determining the variability of both time to onset and duration of BoNT efficacy; (ii) age in particular is a strong predictor of time to onset, with older patients showing an earlier BoNT effect; and (iii) type of BoNT, dosages, and the underlying clinical condition are the main predictive factors of duration of BoNT efficacy. In conclusion, we found novel potential predictors of BoNT efficacy worthy of being assessed in future studies and during clinical practice.

## Supplementary Information

Below is the link to the electronic supplementary material.

Supplementary file1 (DOCX 13 KB)

## Data Availability

The data that support the findings of this study are available from the corresponding author upon reasonable request.
