# Supplementary material for: Time to onset and duration of botulinum toxin efficacy in movement disorders
Source: J Neurol. 2022 Feb 3;269(7):3706–12. doi: 10.1007/s00415-022-10995-2 (PMC9217780; doi:10.1007/s00415-022-10995-2)
Supplement: Supplementary file 1 — Supplementary file1 (DOCX 13 KB) [file 415_2022_10995_MOESM1_ESM.docx]

**Supplementary material: Questionnaire on previous botulinum toxin treatment**

1. For which indication are you currently in treatment with botulinum toxin? When was it first diagnosed?
2. For how many years have you been treated with botulinum toxin?
3. How many days did it take for you to notice the first improvements in your condition regarding your previous treatment?
4. Regarding your previous treatment, how many days did it take for you to feel the botulinum toxin’s effect was wearing off?
